# Using high-frequency data to quantify how urban nature affects air temperature and energy consumption

**DOI:** 10.1073/pnas.2619288123

**Published:** 2026-11-02

**Authors:** Baolong Han, Tong Wu, Stephen Polasky, Zhengwu Cai, Xuelian Pan, Haoqi Wang, Gretchen Daily, Zhiyun Ouyang

**Affiliations:** ^a^ State Key Laboratory of Regional and Urban Ecology, Research Center for Eco-Environmental Sciences, Chinese Academy of Sciences, Beijing 100085, China; ^b^ Natural Capital Alliance, Woods Institute for the Environment, Doerr School of Sustainability, Stanford University, Stanford, CA 94305; ^c^ Department of Applied Economics, College of Food, Agricultural and Natural Resource Sciences, University of Minnesota, St. Paul, MN 55108; ^d^ Department of Ecology, Evolution and Behavior, College of Biological Sciences, University of Minnesota, St. Paul, MN 55108; ^e^ Department of Biology, Woods Institute for the Environment, Stanford University, Stanford, CA 94305

**Keywords:** urban greenspace, nature-based solutions, climate change, low-carbon development, energy savings

## Abstract

Globally, cities account for 78% of energy consumption and 70~75% of greenhouse gas emissions. Using spatially explicit data on urban vegetation, water bodies, and buildings, combined with extensive daily observations of air temperature and electricity consumption for the subtropical megacity of Shenzhen, China, this study shows that urban nature contributes to urban cooling, reducing energy consumption and associated greenhouse gas emissions. We found that green and blue spaces in the city’s built-up area had a pronounced influence on air temperature, ranging from a cooling effect of 1.27 °C to a warming effect of 0.29 °C, with the cooling effect being dominant over the course of a year. The cooling effect was greatest at moderate temperatures (~20 °C) and was lower on hotter days. We identified a temperature threshold of 16 to 18 °C above which urban nature produces measurable reductions in cooling energy demand. We found that residential electricity consumption was more temperature-sensitive than nonresidential electricity consumption. For the study year of 2021, the net electricity savings from urban nature was 1.5 billion kWh. This reduction in energy consumption translated to almost one million tons of avoided CO_2_ emissions, which was 4.65-times the sequestration capacity of the city’s terrestrial vegetation and equivalent to the annual emissions of over 223 thousand people using global average annual per capita carbon emissions.

## Significance

This study used high-frequency and high-resolution data to quantify the relationship between urban nature, air temperature, and energy consumption in Shenzhen, China. On most days, urban nature reduced ambient air temperature, with the maximum cooling occurring with temperatures ~20 °C. In 2021, the net electricity savings attributable to urban nature was 1.5 billion kWh, leading to a reduction of almost one million tons of CO_2_ emissions. Incorporating urban nature into city- and neighborhood-scale planning can provide cooling benefits to densely populated communities with high heat exposure and corresponding energy demand.

The measurement of urban ecosystem services makes explicit the benefits that nature provides to city residents (1, 2). One important urban ecosystem service is local climate regulation (3, 4). Vegetation and water bodies in urban areas can lower ambient air temperatures thereby mitigating the urban heat island effect reducing energy use and associated greenhouse gas emissions (1, 4, 5). Quantifying this ecosystem service involves two key steps: 1) measuring how urban nature changes ambient air temperature; and 2) measuring how changes in ambient air-temperature affect energy use. Here we use high-frequency high-spatial resolution data on the location of urban nature, buildings, air temperature, and electricity use to quantify the effect of urban nature on temperature, energy use, and greenhouse gas emissions in Shenzhen, China, a subtropical coastal megacity of approximately 18 million people.

While there is a large and rapidly growing literature on various aspects of the urban heat island effect (6–10), providing an integrated framework that directly connects how urban nature’s cooling effect influences energy use requires four requirements that are rarely met in existing studies. First, the analysis should focus on the effects of urban nature on air temperature rather than land surface temperature because the former affects heating and cooling directly. While land surface temperature is easier to obtain (11), studies have found that land surface temperature differs substantially from the corresponding air temperature (4, 12–14). Air temperature is therefore a more appropriate indicator for evaluating the cooling effect of urban nature (4, 7, 9, 14–16). Langemeyer et al. (17) identified the limitations of using land surface temperature data to assess urban thermal conditions. They therefore used air-temperature data obtained through mobile measurements to construct a spatial raster of average air-temperature differences across Barcelona, which was then used to assess the potential thermal-regulation benefits of green roofs (17). Second, the analysis should include the impact of different types of natural spaces across the urban area rather than focusing only on parks or green roofs (8, 17, 18). Masoudi et al. (19) noted that 65% of relevant studies examined only one type of urban nature (19). Third, the analysis should account for seasonal variation in the cooling effect (20). Existing studies have largely focused on summer (4, 14, 19, 21, 22), whereas year-round analysis is needed to capture differences across seasons (15, 23, 24). Zhao et al. (21) found that lakes and their surrounding green spaces at Wuhan, China produced a cooling effect with substantial seasonal variation. Specifically, the cooling effect reached 1.0 °C in summer within a 300 m buffer and was as low as 0.3 °C in winter within a 200 m buffer (21). Finally, estimates of nature-based cooling and changes in energy use should correspond in both space and time. This is perhaps the most difficult requirement. Existing assessments of the effects of urban nature on energy use often rely on parameters taken from other studies conducted during different periods or even in different locations (1, 25). Wang et al. (26) noted that although mesoscale meteorological models can quantify cooling at the urban scale, parameters used to evaluate energy consumption are often derived from building- or neighborhood-scale case studies, resulting in considerable uncertainty in city-scale estimates (26). Based on our review of the existing literature, we did not find a prior study addressing all four requirements simultaneously. The study that comes closest is Grilo et al. (20), which investigated the cooling effects of Mediterranean urban parks (20). This study used air temperature rather than land surface temperature, considered multiple types of green and gray urban spaces, and examined both summer and winter conditions. However, it did not analyze how the cooling effect affected energy use.

To address these requirements, this study focuses on Shenzhen, a coastal megacity of approximately 18 million people in China. Using observations from 237 weather stations, we produced fine-scale daily air-temperature maps for 2021 and combined them with daily spatially- and temporally-matched maps of vegetation, water bodies, and impervious surfaces at a 10 m resolution. We then estimated the daily relationships between air temperature and the composition of urban natural spaces to quantify daily cooling effects. Finally, using daily spatially- and temporally-matched data on citywide electricity consumption, including overall as well as residential electricity use, we assessed how these daily cooling effects translated into energy savings and avoided CO_2_ emissions. This study provides an analytical framework for identifying the contributions of urban nature to cooling, energy saving, and emissions reduction. A defining feature of this study is its quantification of links between urban nature, air temperature, and electricity consumption for an entire city using temporally high-frequency (daily observations) and spatially high-density (of meteorological measurements) data.

## Results

### Urban Nature’s Cooling Effect.

**Fig. 1.**
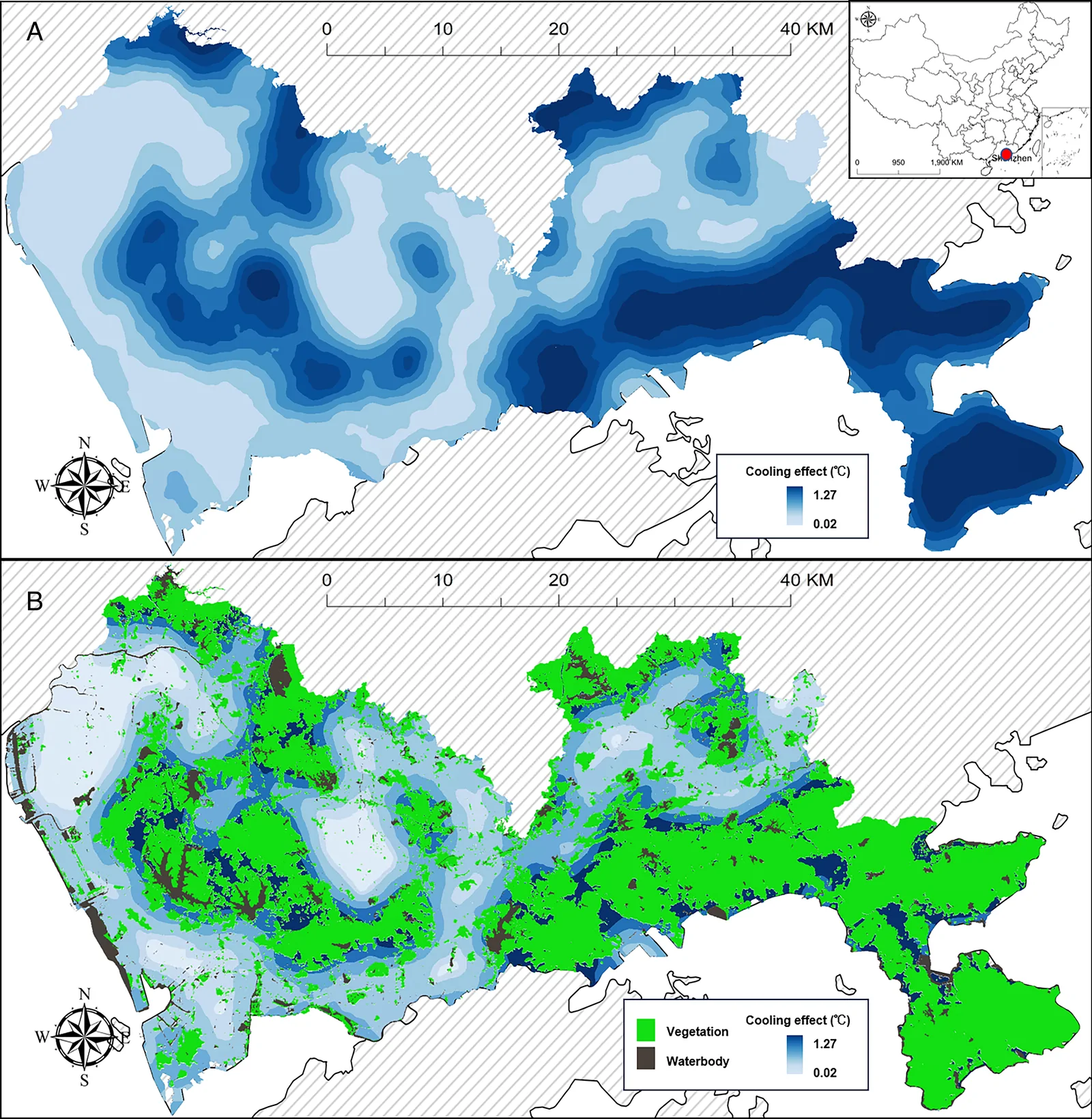
The spatial distribution of average daily cooling effect in Shenzhen (*A*) and its built-up area (*B*), showing a tight relationship with urban natural space. This chart is created by averaging and overlaying daily temperature drop maps. See *Materials and Methods* for more details.

**Fig. 2.**
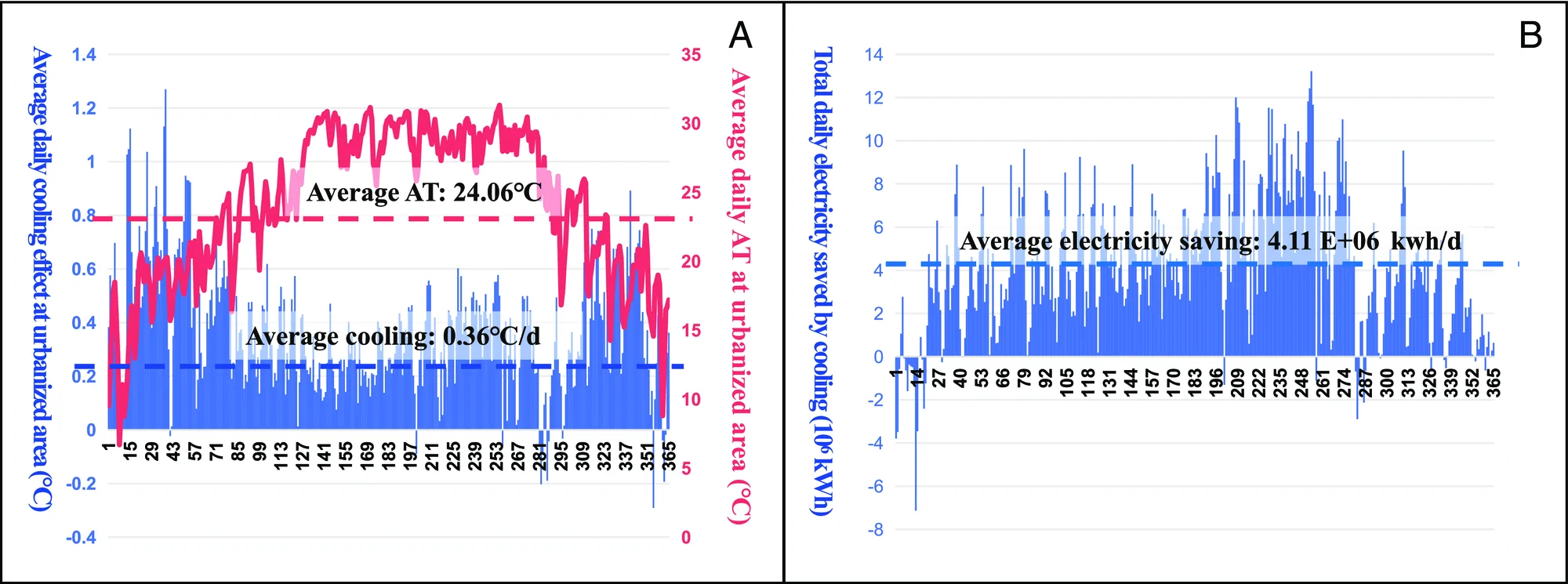
The daily average cooling effect of urban nature area and its impact on electricity consumption in Shenzhen’s built-up area in 2021. (*A*) There is a greater cooling effect at moderate temperatures than at high temperatures. (*B*) Daily electricity consumption is reduced by the urban nature cooling effect. The electricity usage gap between air temperature with and without the cooling effect is the electricity saved through urban nature cooling (see *Materials and Methods*).

In 2021, the greenspaces and water bodies of Shenzhen’s urban area contributed to the regulation of air temperature ranging from an overall city cooling effect of 1.27 °C (Feb. 7th, 2021) to a warming effect of 0.29 °C (Dec. 27th, 2021)—with the cooling effect being dominant when aggregated across the year (Figs. 1*B* and 2*A*). There was a strong positive relationship between the average daily cooling effect of each pixel and the proportion of greenspace within a 2,500 m buffer area of the pixel. On average, a 30% coverage of green space within a pixel’s buffer area was correlated with 0.38 °C cooling in its daily air temperature over the whole year (Fig. 1).

The strongest effect of nature-based cooling occurred at moderate temperatures (around 20.19 °C) and weakened at higher temperatures (Fig. 2*A*). During the winter (December to February), which had an average air temperature of 17.18 °C, the average cooling effect was 0.49 °C. During the summer (June to August), which had an average air temperature of 26.32 °C, the average cooling effect was 0.32 °C. This finding is consistent with an earlier, coarser study of Shenzhen showing higher cooling effects under cooler air temperatures (27).

### Urban Nature’s Contribution to Reduced Electricity Usage.

**Fig. 3.**
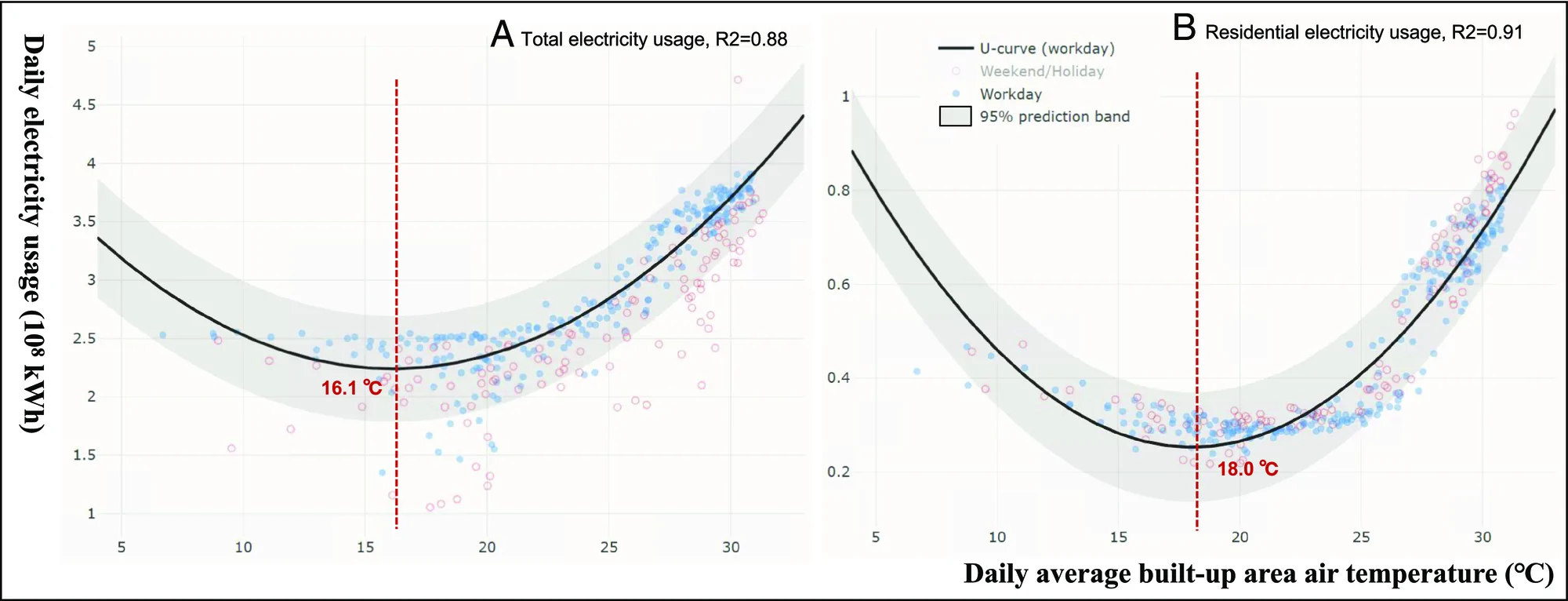
The marginal change of total electricity (*A*) and residential electricity (*B*) consumption under different daily average air temperature shows the relationship between air temperature within built-up area and electricity consumption during workdays. Most outliers were days near major holidays. Since the differences between formulas for different day types are solely reflected in the coefficients of dummy variables—resulting only in variations in the curve intercepts—their temperature inflection points remain identical.

In Shenzhen, the average daily net electricity savings from urban nature’s cooling effect in 2021 was 4.11 million kWh/d (Fig. 2*B*). The highest daily savings was 13.22 million kWh/d on Sep. 11, 2021, and the lowest was −7.13 million kWh/d on Jan. 13, 2021, when there was increased demand for heating. The electricity consumption during the warmest workday on July 23 (average urban air temperature of 30.91 °C, Fig. 3*A*) decreased by 7.53 million kWh/d, while during the coldest workday of Jan. 8 (average urban air temperature as 6.69 °C) the electricity consumption change was −1.60 million kWh.

For the entire year, the annual net electricity savings from the urban nature’s cooling effect amounted to 1501.27 million kWh/yr, which is equal to 1.46% of total energy consumed in 2021. The annual net electricity savings included the total energy saving from cooling of 1541.05 million kWh/yr and a total increase in energy consumption from heating of 39.78 million kWh/yr. The threshold air temperature below which further cooling led to an increase in electricity use was 16.1 °C (turning point of Fig. 3*A*). Below this temperature, people and industry require more heating than cooling and lower temperatures leading to increased energy use.

Residential electricity consumption showed greater sensitivity to temperature than did nonresidential energy consumption. A 1 °C rise in daily air temperature was associated with an increase of 1508.03 million kWh/yr in residential consumption and an increase of 4631.41 million kWh/yr in total consumption. The increase in residential consumption from a 1 °C rise in daily air temperature was 32.56% of the total consumption increase, which is higher than its baseline share of 16.68% of total electricity consumption. The net energy savings from the cooling services provided by urban nature to residential electricity consumption amounted to 472.24 million kWh/yr, equivalent to 2.75% of the annual residential electricity consumption.

Beyond its greater temperature sensitivity, residential electricity shows a day-type response that is the reverse of total consumption. The weekend effect is positive for residential use (+3.89 million kWh/d, *P* < 0.001) but negative for total consumption (−22.4 million kWh/d), reflecting a workday-to-weekend shift from industrial/commercial to residential demand. Relatedly, the spring-festival and holiday effects that are strong in the total model are not significant for residential use. (See *SI Appendix*, *sections S1* and *S2*.) This contrast indicates that urban nature’s cooling benefit accrues disproportionately to the residential sector, whose electricity use profile differs from the citywide aggregate.

### Electricity Usage and CO_2_ Emissions Reduction.

The annual net electricity savings from urban nature’s cooling effect accounted for 1.46% of Shenzhen’s total annual electricity consumption (102.95 billion kWh) in 2021. Shenzhen’s CO_2_ emissions parameter for electricity generation was 0.6379 kgCO_2_/kWh (28), so this saving is equivalent to a net reduction of 0.96 million tCO_2_ in annual emissions. This accounted for approximately 3.84 percent of the city’s annual CO_2_ emissions quota, a municipal regulation that caps emissions at 25 million tons and covers 750 industrial entities (29). Furthermore, this avoided emissions was equivalent to annual CO_2_ emissions of 223,255 people as measured by the global average of 4.3 tCO_2_/capita/yr (30).

## Discussion

Using spatially explicit data on the location of urban nature and buildings, daily air-temperature data from 237 weather stations and daily electricity-consumption data, this study quantified the daily cooling, energy saving, and CO₂ emissions reduction effects of urban nature in Shenzhen. Urban nature reduced air temperature by an average of 0.49 °C in winter and 0.32 °C in summer, yielding annual net electricity savings of 1.50 billion kWh and avoided CO₂ emissions of 0.96 million tons.

Results showed that maximum cooling from urban nature occurred around 20 °C. As temperature increases beyond this point, high vapor-pressure deficit (VPD) and soil-moisture limitations can induce stomatal closure, suppressing evapotranspiration and weakening cooling (7). In subtropical Shenzhen, temperatures in the range of the cooling optimum occurs in late autumn to early winter (November to January). This perspective may explain the difference between previous studies and our findings. In contrast to our findings of greater average cooling in late autumn and winter, a study of five European cities found the cooling effect from greenspaces was higher in summer (31), when these cities were closer to the cooling optimum. The contrast may be further amplified by vegetation phenology: Shenzhen has evergreen vegetation compared to the deciduous vegetation that prevails in more northern cities. More broadly, cooling from greenspace is shaped not only by ambient temperature but also by solar radiation, surface heat storage, wind conditions, irrigation, and the size, composition, and spatial configuration. During heat waves, excessive heat input may exceed urban nature’s cooling capacity, resulting in smaller near-surface temperature reductions than on milder days.

Vegetation cooling had a statistically significant effect on 93.7% of observed days (342/365). The 23 nonsignificant days (6.3%) and 8 warming days (2.2%, any significant) were driven almost exclusively by cold, humid, and overcast conditions, with lower mean temperature (2.8 °C), increased humidity (14.9 percentage points), and reduced sunshine (−4.4 h) compared to the annual average. On these types of days, plant transpiration was suppressed and canopy insulation largely offset evaporative cooling. Water-body cooling was significant on 57.3% of the observed days (209/365). However, on 108 d waterbodies had a warming effect (any significance), which was concentrated in the months of October to December. These autumn-winter warming days were characterized by higher atmospheric pressure (+1.4 hPa), lower ground temperature (−2.7 °C), and reduced sunshine (−1.3 h) compared to the annual average, conditions under which water bodies, with their high thermal capacity (two to three times that of soil), release stored summer heat to the surrounding atmosphere while the land cooled more rapidly, producing a “thermal inertia” effect. The remaining 156 d (42.7%) were nonsignificant, occurring predominantly in transitional seasons when the land–water temperature gradient was too weak to yield a detectable signal in either direction. Although the daily vegetation coefficient can vary markedly according to meteorological conditions, the annual energy-saving estimate is robust to this variation: replacing the 365 daily coefficients with the year-mean coefficient changes the annual cooling total by less than 1%.

Cooling does not always lead to energy savings in Shenzhen, as the threshold where cooling led to an increase in overall electricity use was 16.1 °C, and 18.0 °C for residential electricity use. Other studies found higher temperatures at which cooling electricity usages show a significant upward trend: 18.94 °C in Beijing and 19.54 °C in Shanghai (32). This difference may stem from variations in perceived temperature caused by differences in humidity and wind speed across cities, as well as by variations in electricity consumption patterns due to different industrial and population structures, which in turn affect temperature sensitivity (33). Notably, Shenzhen has substantially higher average relative humidity (74%) than Beijing (56%) in 2021 (34, 35), resulting in higher perceived temperature at a given air temperature and, consequently, a lower threshold at which cooling becomes necessary. Shenzhen also had a lower average wind speed (1.9 m/s) than Shanghai (2.3 m/s) in 2021 (36), which further elevated perceived temperatures and lowered the cooling threshold. Moreover, Shenzhen’s temperature threshold for total electricity consumption was lower than that for residential electricity consumption, suggesting an even lower threshold in nonresidential areas. Studies conducted in the United States (37), Hong Kong (38), and United Arab Emirates (39) have shown that in nonresidential buildings such as office buildings and shopping malls, air-conditioning set-point temperatures are often lower than those in residential buildings. These studies utilize different analytical methods and datasets, so the comparison of findings may reflect methodological difference in addition to climatic and structural factors.

The electricity saved through the cooling effect of Shenzhen’s urban nature was equivalent to a net reduction of 0.96 million tons of CO_2_ emissions in 2021. This amount is 4.65-times the total volume of carbon sequestered by all terrestrial greenspace in the city (206,600 tons) (40). Although carbon sequestration and avoided emissions are distinct climate benefits that are not directly additive in carbon accounting, urban nature in this study delivers both—cutting emissions by lowering energy demand and storing carbon as a terrestrial sink (41).

Urban nature provides benefits beyond energy saving and the corresponding reduction of carbon emissions. Since the global urban population will be increasingly exposed to extreme heat (42), urban nature’s cooling effect also reduces heat-related illnesses and deaths (43). For instance, if tree cover increased by 10% in the US city of Phoenix, its mortality could decrease by 22% (44). In addition to cooling, urban nature also provides ecosystem services such as air purification (45, 46), runoff retention (47), coastline protection service (48), recreation and health services (43, 49–51), among other benefits (52, 53).

One limitation of this study is the use of the average carbon emissions factor from electricity generation (0.6379 kgCO₂/kWh) published by the Department of Ecology and Environment of Guangdong Province. Ideally, the analysis would use marginal emissions factors derived from power dispatch data, but such information was not available. The empirical relationship between air temperature and electricity consumption is shaped by each city’s specific climate, geography, and industrial structure, precluding direct cross-city comparisons. Nonetheless, while the quantitative results of this study may not transfer to cities with substantially different conditions, the methodological framework—combining spatial buffer regression with quadratic daily urban temperature-daily electricity consumption modeling—remains applicable as a general approach to quantify the urban cooling and energy consumption effects of urban nature elsewhere.

A further limitation is that wind speed and humidity were excluded from the daily temperature regression analysis because these variables were observed only at station locations and cannot be reliably spatialized. At the same time, omitting them avoids propagating interpolation error into the subsequent temperature mapping (*Materials and Methods*). Their omission, however, means that the daily vegetation and water-body coefficients may have absorbed part of the spatial variation that wind speed and humidity would otherwise explain. Finally, it should be noted that meteorological conditions vary across years, yet mean air temperature, precipitation, wind speed, and relative humidity at 2021 was assessed against a 10-y Shenzhen climatology (2016–2025) and fell within the normal range (2 SDs of their climatological means), so it is a climatologically representative rather than anomalous year; the energy-saving estimate nevertheless remains a single-year figure and could differ under other annual conditions.

Our findings strongly suggest that urban nature should be more explicitly considered in land use zoning (54), energy management, and climate policy (55). Urban planning and design could also move beyond a narrow focus on the amount of greenspace to consider its spatial configuration and ecosystem services provision. The estimated optimal influence radius (2,500 m in Shenzhen) found in this study highlights the relevance of neighborhood-scale planning and suggests that connected green–blue networks may help extend cooling benefits to densely populated communities with high heat exposure and energy demand. Furthermore, the electricity savings and avoided indirect emissions provided by urban nature could be incorporated into cost–benefit analysis, carbon accounting, and green investment decisions. The findings from this study provide direct evidence for better urban planning, design, and management.

## Materials and Methods

### Research Design.

This study develops an analytical framework linking natural spaces, air temperature, and electricity consumption using high-frequency and high-resolution data. Using daily observations of air temperature from 237 meteorological stations and electricity use for each day in 2021, we identify the daily cooling effects of vegetation and water bodies, and evaluate the electricity-saving benefits.

First, daily average air temperature at 237 meteorological stations was used as the dependent variable, while surrounding vegetation coverage, water-body coverage, mean elevation, and distance to the coastline were used as explanatory variables. Separate regression models were estimated for each of the 365 d. Wind speed and humidity were excluded because they were only available at station locations and could not be reliably spatialized, which would introduce uncertainty into subsequent temperature mapping. By comparing model performance across buffer radii ranging from 100 to 4,000 m, we chose the buffer radius with the best fit (highest overall R^2^ & mean R^2^). This procedure indicated that R^2^ peaked at ~2,500 m before declining, so a 2,500 m buffer was identified as the optimal spatial extent of natural-space effects on air temperature. See the *SI Appendix*, *section S3*.

Second, based on daily land-cover data from Google Earth Engine Dynamic World (GDW), vegetation coverage, water-body coverage, DEM, and distance to the coastline were extracted for the 2,500 m neighborhood surrounding each 10 m pixel in Shenzhen. These spatial variables were then incorporated into the 365 daily regression equations to generate daily temperature maps for the entire year. A counterfactual scenario in which vegetation and water-body coverage were both set to zero was further developed to simulate air temperatures in the absence of natural spaces, thereby quantifying the cooling contribution of natural spaces within built-up areas.

Finally, daily average temperature in built-up areas was used to explain daily total electricity consumption through an OLS model incorporating linear and quadratic temperature terms, as well as dummy variables for public holidays, weekends, and compensatory workdays. By entering built-up-area temperatures under the observed and no-natural-space scenarios into this model, the daily electricity savings attributable to the cooling effects of natural spaces can be estimated.

### Study Area: Shenzhen.

**Fig. 4.**
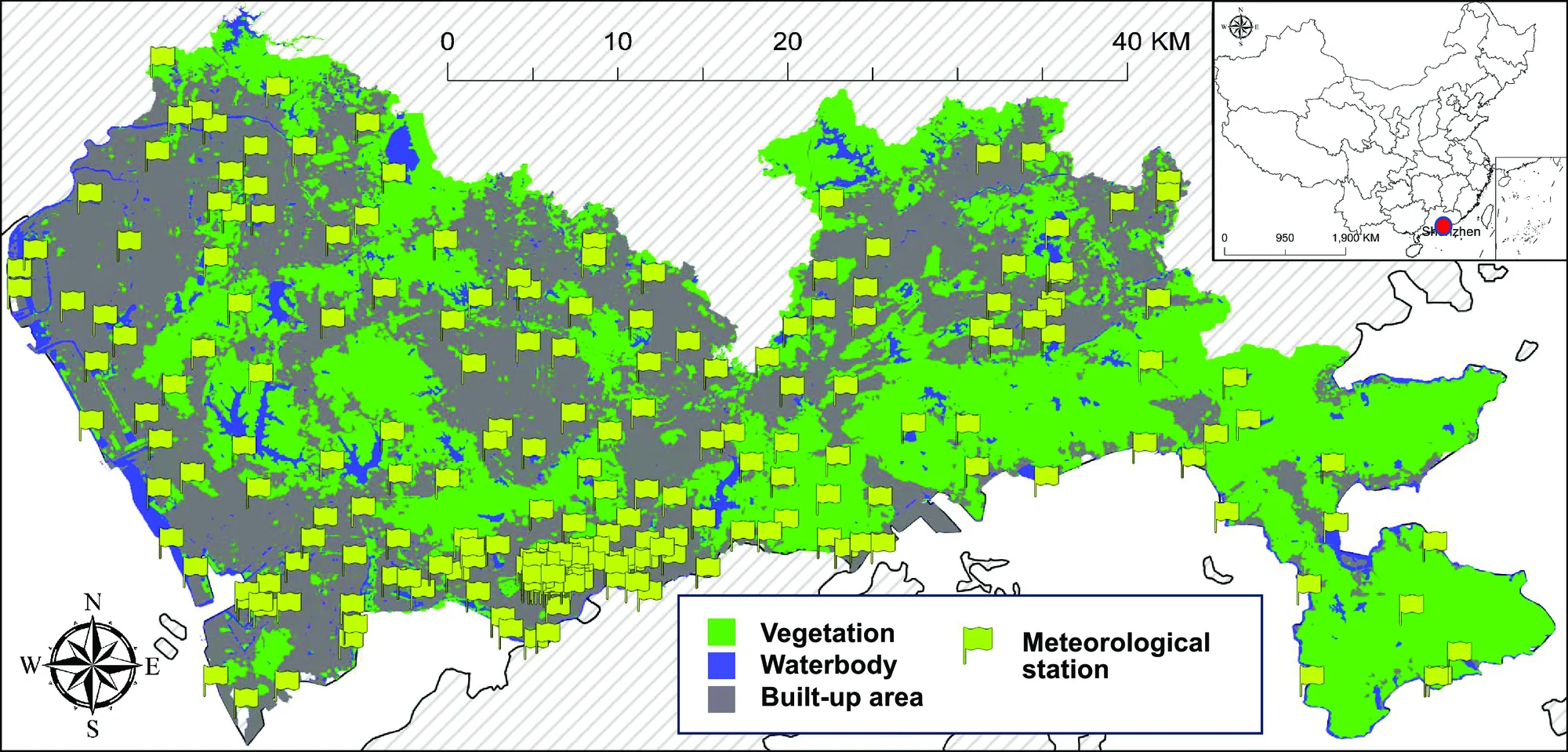
Shenzhen and its land cover. The built-up area covered 51.3% of the total city jurisdiction. Therefore, this area can be considered the population agglomeration zone and the primary area of electricity consumption. This map was prepared using the annual daily land cover mode values.

This study focuses on the city of Shenzhen, which is in the southern coastal region of China and shares borders with Hong Kong and Guangzhou (Fig. 4). It has an average air temperature of 24.22 °C. Over the past three decades, it has been one of the fastest urbanizing areas in China. In 2021, Shenzhen had a population of 18 million and a GDP of 3.07 trillion RMB in an area of approximately 2,000 km^2^. Just over half of land area (50.94%) is built-up surface (measured at 10-m resolution).

As electricity consumption was assumed to occur only within built-up areas, this study considered only natural spaces that could produce significant cooling effects to the built-up area—namely, natural spaces located within a 2,500 m buffer outside the built-up-area boundary.

### Data Sources.

The air-temperature dataset comprises daily mean temperatures for 2021 collected from 237 local meteorological stations. After removing invalid records, an average of 217 stations provided valid observations per day. The multitemporal land cover data was obtained from GDW for the year 2021 with a 10 m resolution (56). We applied a gap-filling procedure using the most recent available observation prior to each missing date to obtain daily land cover data (57). We reclassified land cover data into three categories: vegetation (original classes 1, 2, 4, 5), water (original classes 0, 3), and other (impervious surfaces and bare ground, classes 6 and 7; there is no snow/ice class 8 in Shenzhen). We used the Copernicus GLO-30 global digital elevation map (DEM), which was resampled to 10 m resolution. The electricity consumption data comes from Shenzhen’s electricity statistics table and represents daily electricity usage in 2021. Electricity use for each user is metered by the utility so that data on residential and nonresidential energy use are available from the utility.

### Regressing Air Temperature and Natural Spaces.

We assumed that a pixel’s temperature (dependent variable) was influenced by factors within its surrounding area (independent variables), such as land cover type, altitude, distance from the coastline, vegetation quality, wind speed, humidity, and background temperature. Since the subsequent spatialization process requires these independent variables to be spatially represented, and we only have station-based data for wind speed and humidity, making accurate spatial interpolation impossible, we had to exclude wind speed and humidity as independent variables to reduce uncertainty in subsequent spatial predictions. To account for daily variations in background temperature and other factors, we applied a daily regression approach.

To determine an appropriate impact range, we constructed hybrid buffer scenarios using buffer zones with radii ranging from 100 m to 4,000 m (at 100-m intervals) and selecting those with the best fit (highest overall R^2^ & mean R^2^). A total of 14,600 linear regression models were estimated after excluding 7,402 observations with missing data. The dependent variable was the daily mean air temperature recorded at meteorological stations. The independent variables included vegetation proportion, water-body proportion, and cumulative Enhanced Vegetation Index (EVI) within each buffer zone, as well as station elevation (DEM) and distance to the coastline. Among the 237 stations analyzed, a multicollinearity test revealed strong collinearity between vegetation proportion and EVI, leading to the exclusion of EVI. Ultimately, by comparing the overall and mean R^2^ across 365 regression models for different buffer zones, the 2,500-m buffer zone demonstrated the highest overall R^2^ & mean R^2^. More details are provided in *SI Appendix*, *section S1*.

Under the 2,500-m buffer scenario, the mean adjusted R^2^ over 365 d was 0.75, with all results being statistically significant according to the F-test. Specifically, vegetation coverage was significant (*P* < 0.05) on 342 d (see the *SI Appendix*, Figs. S1 and S2), consistently demonstrating a cooling effect; the temperature reduction at 10% vegetation coverage ranged from [−0.44, −0.03]. Water body coverage was significant on 209 d (including 38 d exhibiting a warming effect), with a temperature reduction at 10% coverage ranging from [−0.29, 0.26]. Vegetation and water body coverage were both nonsignificant on only 5 d. DEM and distance to coastline showed significance on 99.2% and 71.2% of the dates, respectively. Daily diagnostics showed that 57% of the 365 regressions passed the Jarque-Bera test at α = 0.05, with a median *P* of 0.096. The mean skewness was −0.19 and the mean excess kurtosis was +0.56, indicating that the residual distributions were approximately normal. Each daily regression included approximately n ≈ 217 observations, well above the commonly used threshold of 30 for large-sample inference. Therefore, although 43% of the daily models did not pass the Jarque-Bera test, according to the central limit theorem, the *t* tests for coefficient estimates and overall F-tests remain asymptotically valid, and the reliability of model inference is not materially affected. The direction and significance of the relationships between vegetation and water-body coverage and air temperature are discussed in both the *Discussion* section and the *SI Appendix*, *section S4*.

### Mapping Air Temperature With and Without Natural Spaces.

Using the GDW dataset, we generated daily land cover data (with the most recent available data when unavailable). To enhance classification accuracy, land cover types were categorized into three classes: vegetation, water bodies, and built-up areas. Subsequently, we utilized 10-m-resolution raster pixels across Shenzhen to extract daily vegetation coverage (365 images), daily water body coverage (365 images), DEM (1 image), and average distance to coast (1 image) within a 2,500-m buffer zone.

Finally, using the 365 formulas obtained in the previous step and these mapped data, a daily temperature distribution map for each of the 365 d can be generated. By applying the counterfactual assumption of zero vegetation coverage and zero water body coverage, the temperature values under no natural spatial conditions can be determined.

To validate the effectiveness of this approach, we conducted 10-fold cross-validation (CV) and signal-to-noise ratio (SNR) analyses (*SI Appendix*, *sections S1* and *S5*). At the daily level, the mean CV R^2^ of 0.686 means that roughly two-thirds of the spatial variance in absolute air temperature on a given day is captured. This statistic describes the prediction of absolute temperature, which enters our analysis only as an intermediate step. The cooling estimate is obtained as the difference between the observed and counterfactual predictions of the absolute temperature using the same regression and therefore the intercept and every location-specific term common to the two scenarios cancels out. The uncertainty of the cooling estimate is confined to the vegetation and water-body coefficients (mean daily SE 0.00197 °C per percentage point; i.e., 0.059 °C for a 30-percentage-point change on a single day (≈0.00197 × 30) and 0.003 °C in the annual mean (≈$0.059/\sqrt{365}$), the SE of the mean over the 365 d of 2021, and at the annual scale the resulting electricity-saving estimate is 22-times its bootstrap SE (95% CI 13.99 to 16.73 × 10^8^ kWh, *P* < 0.0001). Because the CV gap reflects imperfect prediction of the intermediate absolute temperature, which is common to both the observed and counterfactual predictions from the same regression, it cancels when the two are differenced and therefore does not bias or add uncertainty to the cooling or electricity-saving estimates.

The counterfactual of zero natural cover is supported by the observed buffer land cover. Across the 237 station buffers, vegetation coverage ranges from 0.64 to 98.69% (mean 28.21%) and water-body coverage from 0.34 to 62.36% (mean 11.06%). The joint counterfactual further imposes zero water-body cover at the same time. An exact simultaneous zero of both predictors is never observed; however, 155 of the daily station samples have both vegetation and water-body cover below 1%, showing that near-zero joint configurations occur empirically. Vegetation and water-body coverage are only weakly correlated across the 237 buffers (Pearson r = −0.12; VIF = 1.01), so the joint counterfactual does not introduce material collinearity—both coefficients remain individually identified and the total cooling estimate is the sum of two separately estimated effects.

### Regressing Air Temperature and Electricity Consumption.

This study conducted an OLS regression analysis on the relationship between daily average temperature (°C) within built-up area and daily total electricity consumption (in 100 million kWh). The model was specified as a quadratic function incorporating a day-type dummy variable. The dependent variable was daily total electricity consumption (TE), with independent variables including the linear term (*P* < 0.001, UT) and quadratic term (*P* < 0.001, ${\mathrm{UT}}^{2}$) of built-up average temperature, along with four dummy variables: Spring Festival (*P* < 0.001, S), other statutory holidays (*P* < 0.001, H), compensatory workdays (*P* < 0.05, M), and weekends (*P* < 0.001, E), with regular working days serving as the reference group. Compensatory workdays refer to days when work hours are made up during adjacent weekends when a holiday overlaps with working days.

The model was highly significant overall (F = 436, *P* ≈ 0), with an adjusted R^2^ of 0.878 explaining approximately 88% of the variation in daily electricity consumption; all variables were statistically significant at the *P* < 0.05 level, residuals exhibited near-white noise characteristics (DW = 1.039), and coefficient estimation was reliable. Generalization capability was validated through 10-fold cross-validation, yielding a cross-validation R^2^ of 0.873 (compared to 0.880 for the full sample, a decrease of only 0.007). All variable coefficients demonstrated robustness and are suitable for predicting daily electricity consumption. More details are available in *SI Appendix*, *section S1*.

$$TE=4.2271-0.2465\times UT+0.0076\times UT^{2}-1.1046\times S-0.6565\times H-0.2248\times M-0.2236\times E,$$

Whereas *TE* is total daily electricity consumption in Shenzhen (10^8^ kWh); *UT* is built-up area daily mean temperature (°C); *S* is dummy variable for Spring Festival; *H* is dummy variable for other statutory holidays (besides Spring Festival); *M* is dummy variable for make-up workdays/adjusted workdays; *E* is dummy variable for weekends. Except for the coefficient of the “Make-up workdays” dummy variable, whose significance level is *P* = 0.0112, all other predictors and the intercept are highly significant at *P* ≪ 0.01.

By incorporating the daily average temperature of built-up areas with and without natural spaces, corresponding electricity consumption can be predicted, thereby revealing how electricity consumption changes due to natural space cooling service.

## Supplementary Material

Appendix 01 (PDF)

## Data Availability

All data needed to evaluate the conclusions are present in the paper and/or the *SI Appendix*. Publicly shareable data supporting this study have been deposited in Figshare (57). The raw daily electricity consumption data are not publicly available due to their commercial and economic sensitivity and will be provided by the corresponding author(s) upon reasonable request and review of the proposed research use. The raw meteorological station observations were provided by the Shenzhen Meteorological Bureau under a restricted data-use arrangement and are not publicly redistributable; they may be requested from the Shenzhen Meteorological Bureau or the corresponding author upon reasonable request and review of the intended use. To enable replication while protecting these restricted sources, the corresponding author will share derived, de-identified versions of both restricted datasets that are sufficient to reproduce the study‘s main conclusions.
