# Supplementary material for: Using high-frequency data to quantify how urban nature affects air temperature and energy consumption
Source: Proc Natl Acad Sci U S A. 2026 Nov 2;123(45):e2619288123. doi: 10.1073/pnas.2619288123 (PMC13751669; doi:10.1073/pnas.2619288123)
Supplement: Supplementary file 1 [file pnas.2619288123.sapp.pdf]

Supporting Information for

Using high-frequency data to quantify how urban nature affects air temperature and energy consumption

Baolong Han, Tong Wu, Stephen Polasky, Zhengwu Cai, Xuelian Pan, Haoqi Wang, Gretchen Daily, Zhiyun Ouyang

Corresponding Authors:

Stephen Polasky
Email: polasky@umn.edu

Zhiyun Ouyang
Email: zyouyang@rcees.ac.cn

This PDF file includes:

Supporting text
Figures S1 to S2

Supporting Information Text

S1: Multi-Buffer Full-Variable OLS Regression

ATEMP ~ C1_pct + C2_pct + DEM + DisC | Daily x 40 buffers (100-4000m) | All variables forced

Variable definitions: *ATEMP is the dependent variable, denoting the daily mean air temperature at a station (°C); C1_pct and C2_pct are the key explanatory variables, representing the percentage of vegetation and water pixels, respectively, within the circular buffer relative to the total number of pixels in that buffer; DEM is the station elevation (m); DisC is the Euclidean distance from the station to the coastline (m).*

Goodness-of-fit definitions: *Both Mean R^2 and Overall R^2 are goodness-of-fit measures under the daily regression framework, but they differ in how the daily results are aggregated. Mean R^2 is computed by averaging first: Mean $R^2 = (1/365)\ \Sigma_d R^2_d$, where $R^2_d = 1 - SSE_d / SST_d$. One OLS regression is estimated for each day, yielding a single daily R^2, and the 365 daily values are then averaged with equal weights. It answers the question "how much can a typical day explain", giving every day the same weight irrespective of whether the spatial temperature contrast on that day is large or small. Overall R^2 is computed by aggregating first: Overall $R^2 = 1 - \Sigma_d SSE_d / \Sigma_d SST_d$. The residual sums of squares and the total sums of squares are accumulated separately over the 365 days and the division is performed only once, so that the result is SSE/SST-weighted: days with a large spatial temperature contrast (large SST) carry more weight, whereas days with small contrasts barely affect the value. It answers the question "how much of the total variance is explained by the model across all 79,103 station-day observations".*

1. R2 vs Buffer Radius

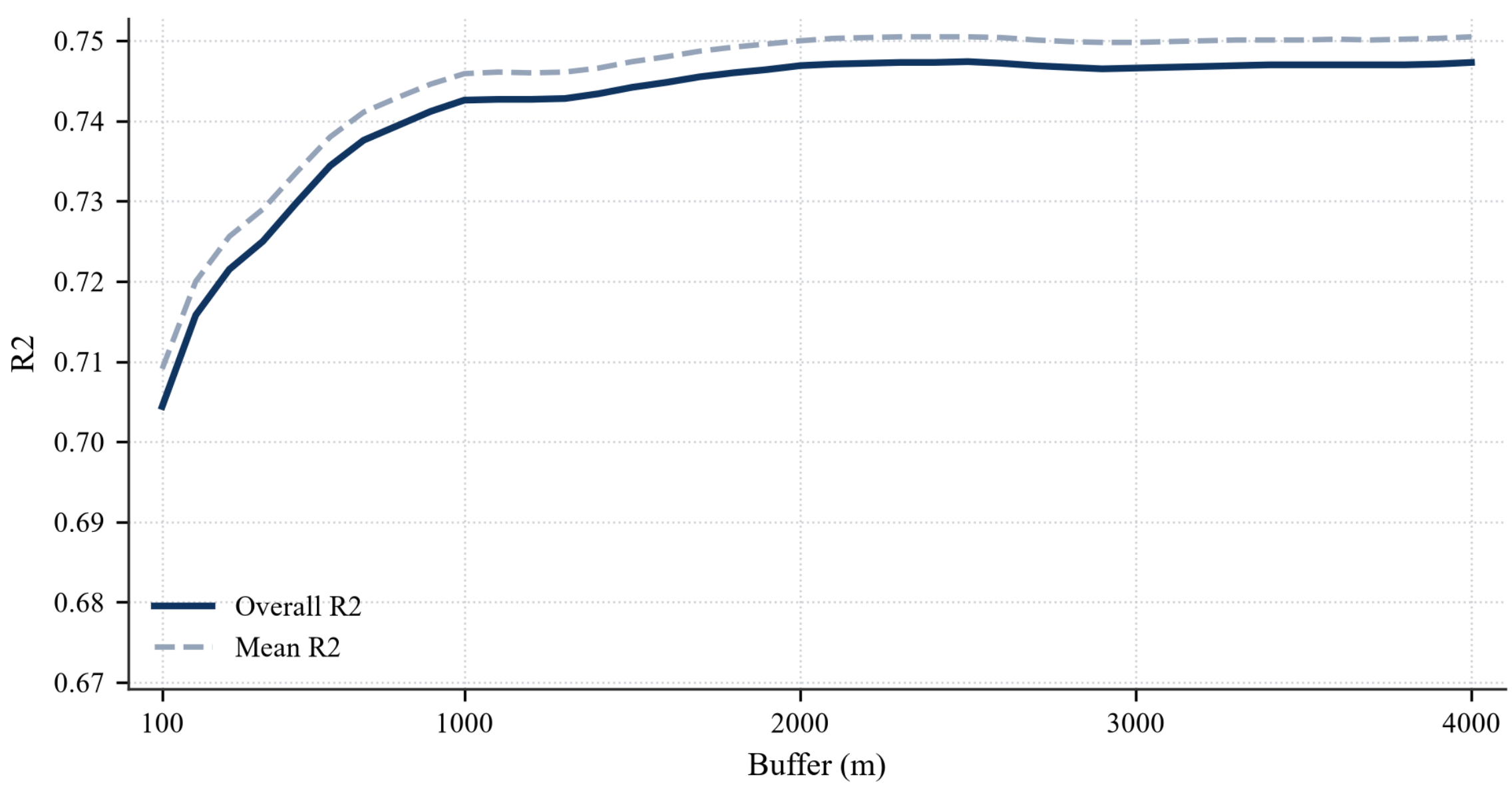

Overall and mean R2 peak at ~2500m, then decline as buffer oversmoothing dilutes local vegetation and waterbody signals.

2. C1_pct / C2_pct Significant Days (%)

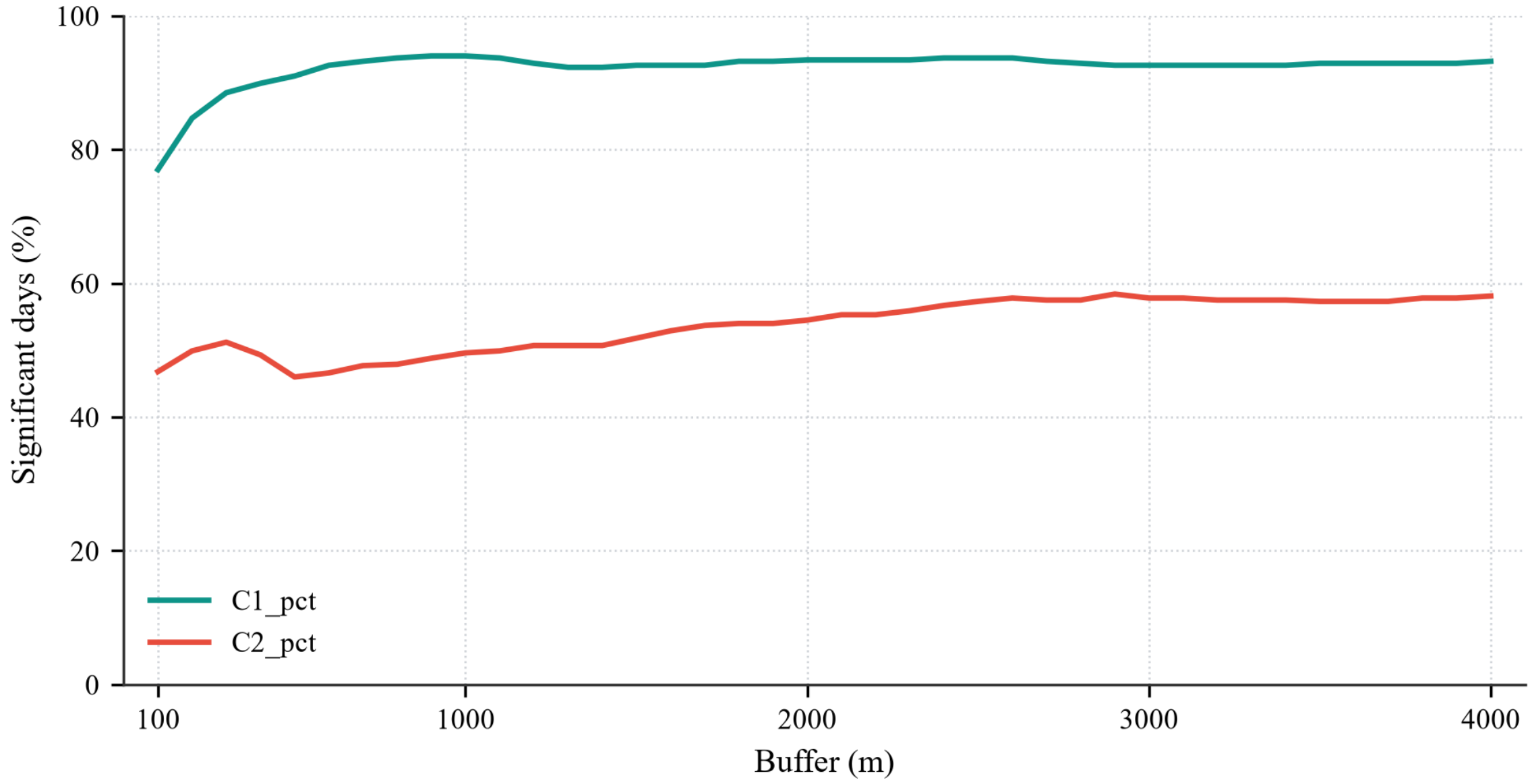

3. DEM / DisC Significant Days (%)

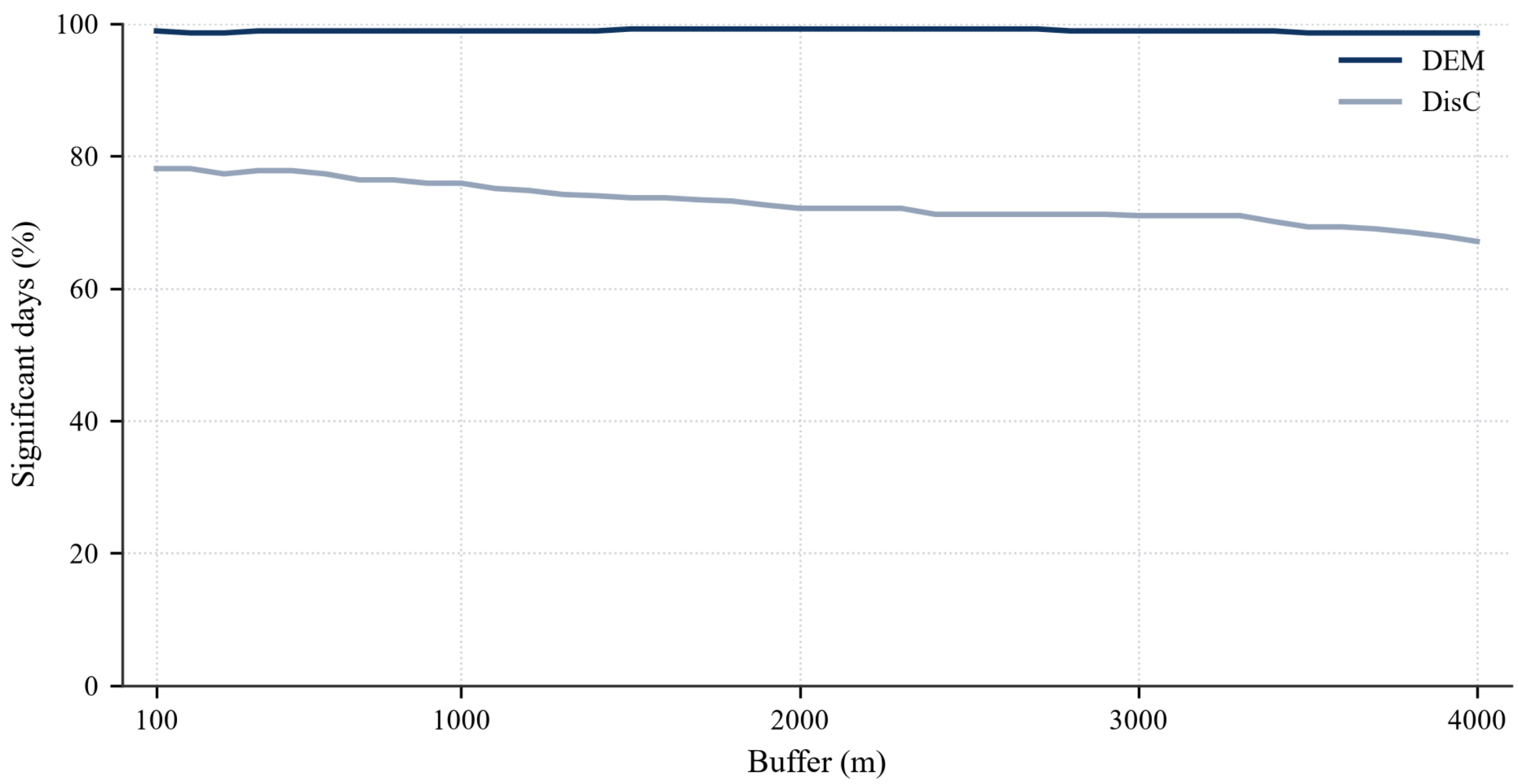

4. Neither C1 nor C2 Significant (%)

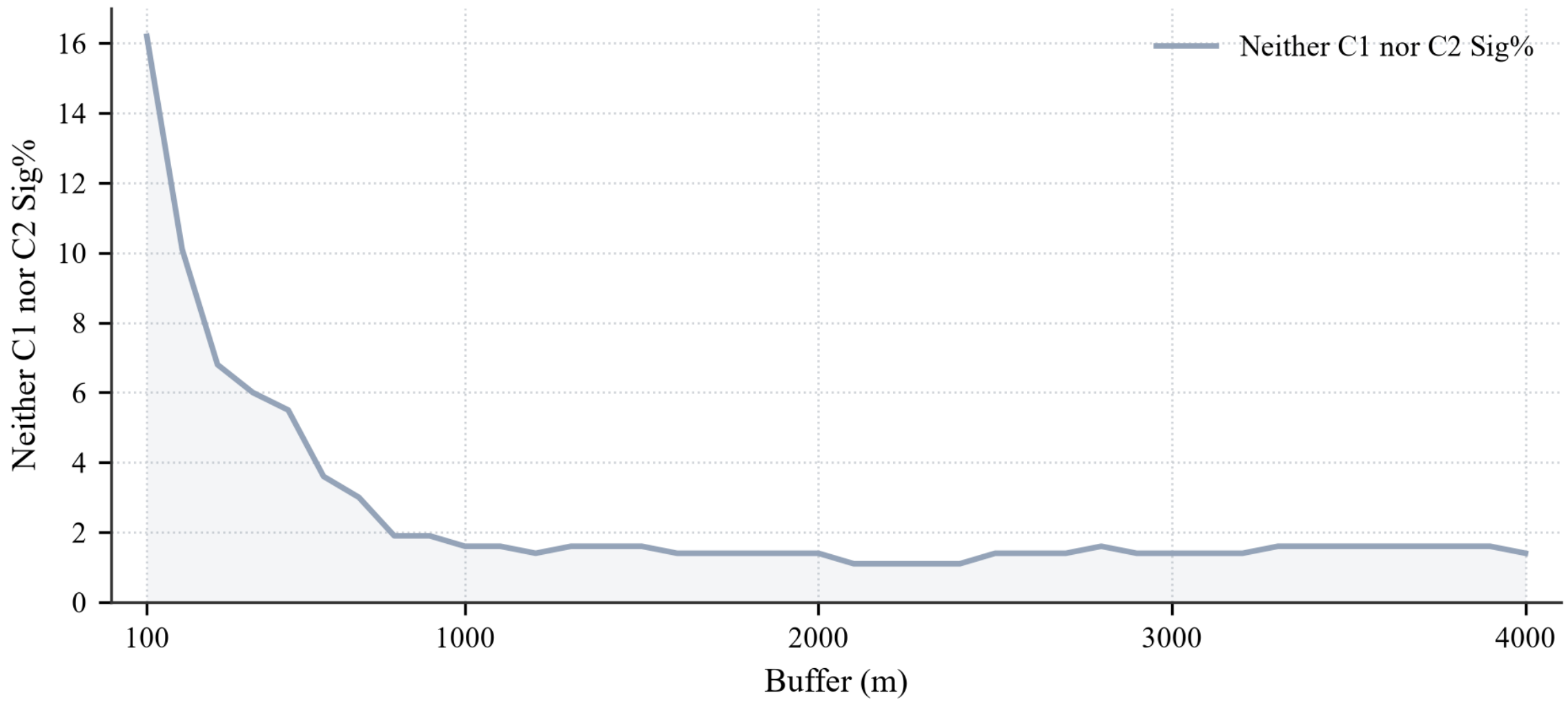

5. Complete Statistics

Buffer (m)	Overall R2	Mean R2	C1_pct Sig%	C2_pct Sig%	Neither Sig	DEM Sig%	DisC Sig%
100m	0.7044	0.7091	77.0%	46.8%	16.2%	98.9%	78.1%
200m	0.7158	0.7200	84.7%	49.9%	10.1%	98.6%	78.1%
300m	0.7215	0.7256	88.5%	51.2%	6.8%	98.6%	77.3%
400m	0.7250	0.7290	89.9%	49.3%	6.0%	98.9%	77.8%
500m	0.7298	0.7336	91.0%	46.0%	5.5%	98.9%	77.8%
600m	0.7344	0.7380	92.6%	46.6%	3.6%	98.9%	77.3%
700m	0.7376	0.7411	93.2%	47.7%	3.0%	98.9%	76.4%
800m	0.7394	0.7429	93.7%	47.9%	1.9%	98.9%	76.4%
900m	0.7412	0.7446	94.0%	48.8%	1.9%	98.9%	75.9%
1000m	0.7426	0.7459	94.0%	49.6%	1.6%	98.9%	75.9%
1100m	0.7427	0.7461	93.7%	49.9%	1.6%	98.9%	75.1%
1200m	0.7427	0.7460	92.9%	50.7%	1.4%	98.9%	74.8%
1300m	0.7428	0.7461	92.3%	50.7%	1.6%	98.9%	74.2%
1400m	0.7434	0.7466	92.3%	50.7%	1.6%	98.9%	74.0%
1500m	0.7442	0.7474	92.6%	51.8%	1.6%	99.2%	73.7%
1600m	0.7448	0.7480	92.6%	52.9%	1.4%	99.2%	73.7%
1700m	0.7455	0.7487	92.6%	53.7%	1.4%	99.2%	73.4%
1800m	0.7460	0.7492	93.2%	54.0%	1.4%	99.2%	73.2%
1900m	0.7464	0.7496	93.2%	54.0%	1.4%	99.2%	72.6%
2000m	0.7469	0.7500	93.4%	54.5%	1.4%	99.2%	72.1%
2100m	0.7471	0.7503	93.4%	55.3%	1.1%	99.2%	72.1%
2200m	0.7472	0.7504	93.4%	55.3%	1.1%	99.2%	72.1%
2300m	0.7473	0.7505	93.4%	55.9%	1.1%	99.2%	72.1%
2400m	0.7473	0.7505	93.7%	56.7%	1.1%	99.2%	71.2%
2500m	0.7474	0.7505	93.7%	57.3%	1.4%	99.2%	71.2%
2600m	0.7472	0.7504	93.7%	57.8%	1.4%	99.2%	71.2%
2700m	0.7469	0.7501	93.2%	57.5%	1.4%	99.2%	71.2%
2800m	0.7467	0.7499	92.9%	57.5%	1.6%	98.9%	71.2%
2900m	0.7465	0.7498	92.6%	58.4%	1.4%	98.9%	71.2%
3000m	0.7466	0.7498	92.6%	57.8%	1.4%	98.9%	71.0%
3100m	0.7467	0.7499	92.6%	57.8%	1.4%	98.9%	71.0%
3200m	0.7468	0.7500	92.6%	57.5%	1.4%	98.9%	71.0%

Buffer (m)	Overall R2	Mean R2	C1_pct Sig%	C2_pct Sig%	Neither Sig	DEM Sig%	DisC Sig%
3300m	0.7469	0.7501	92.6%	57.5%	1.6%	98.9%	71.0%
3400m	0.7470	0.7501	92.6%	57.5%	1.6%	98.9%	70.1%
3500m	0.7470	0.7501	92.9%	57.3%	1.6%	98.6%	69.3%
3600m	0.7470	0.7502	92.9%	57.3%	1.6%	98.6%	69.3%
3700m	0.7470	0.7501	92.9%	57.3%	1.6%	98.6%	69.0%
3800m	0.7470	0.7502	92.9%	57.8%	1.6%	98.6%	68.5%
3900m	0.7471	0.7503	92.9%	57.8%	1.6%	98.6%	67.9%
4000m	0.7473	0.7505	93.2%	58.1%	1.4%	98.6%	67.1%
Mix	0.7430	0.7465	93.7%	53.7%	1.1%	98.9%	71.2%

Note: *Sig% is the share of days on which a variable is statistically significant. For a given buffer radius, 365 daily OLS regressions are estimated (one for each day of the year), and Sig% is the percentage of those 365 days on which the coefficient of that variable is significant at $p < 0.05$ under a two-sided t-test.*

6. Recommended Buffer

Metric	2500m	1000m	2000m	3000m
Overall R2	0.7474	0.7426	0.7469	0.7466
Mean R2	0.7505	0.7459	0.7500	0.7498
C1_pct Sig%	93.7%	94.0%	93.4%	92.6%
C2_pct Sig%	57.3%	49.6%	54.5%	57.8%

R2 peaks at 2500m. Beyond this radius, spatial smoothing degrades the local vegetation and waterbody temperature signal. Recommended analysis window: 2500m.

7. Mixed Buffer: C1(900m) + C2(2900m)

Variable	Source	Metric	Unified 900m	Unified 2900m	Mix
C1_pct	900m (optimal)	Sig%	94.0%	92.6%	93.7%
C2_pct	2900m (optimal)	Sig%	48.8%	58.4%	53.7%
Overall R2	-		0.741	0.747	0.743
Mean R2	-		0.745	0.750	0.747
Neither C1&2	-		1.6%	2.2%	1.1%

Mixed buffer (C1@900m + C2@2900m) overall R2=0.743, between unified 900m (0.741) and 2900m (0.747), not surpassing unified 2500m (R2=0.747).

> **Conclusion:** Full-variable OLS regression confirms a stable vegetation cooling signal across all spatial scales. R2 peaks at 2500m before declining -- beyond this radius, spatial resolution loss outweighs statistical gains. The unified 2500m buffer is recommended as the optimal analysis window.

S2: Meteorological Drivers of the Anomalous Vegetation and Waterbody Effects

2500 m buffer | 237 stations × 365 days | Weather observations: Shenzhen national meteorological station (WMO ID 59493)

1. Overview

In the daily regressions, the coefficient on C1_pct (vegetation cover) is statistically significant at $p < 0.05$ on 93.7% of days (342 of 365). Of these, 339 (92.9%) are negative, i.e. indicative of cooling, and 3 (0.8%) are positive. On the remaining 23 days (6.3%) the C1 coefficient is not significant. Taking sign and significance together, the C1 point estimate is positive on 8 days in all (2.2%), but only 3 of those 8 are statistically significant. The signal for C2_pct (water cover) is much weaker: the coefficient is significant on 57.3% of days (209 of 365), but negative on only 171 of them (46.8%), while 38 days (10.4%) yield a significantly positive coefficient; on 156 days (42.7%) the coefficient is not significant, and the point estimate is positive on 108 days in all (29.6%), of which 38 are significant. This appendix examines the meteorological conditions associated with these non-cooling and apparently warming days, using daily observations from Shenzhen national meteorological station (WMO ID 59493). Throughout, "warming days" refers to days with a positive point estimate irrespective of significance; the subset that is itself statistically significant is reported separately.

> Key conclusion: The C1 coefficient loses significance — and occasionally reverses sign — under cold, wet, overcast conditions (high humidity, little sunshine, heavy precipitation). Apparent warming by C2 is concentrated in the autumn–winter transition (October–December) and is attributable to the large thermal inertia of water: water bodies release the heat stored over summer and are therefore relatively warm compared with the land once the air cools.

2. C1_pct Anomalous Effects

2.1 Summary Statistics

Category	Days	Share	Dominant Months
C1 significant ($p < 0.05$), all signs	342	93.7%	Year-round
— of which negative (cooling)	**339**	**92.9%**	Year-round
— of which positive (warming)	3	0.8%	Oct (2), Dec (1)
C1 non-significant	23	6.3%	May (7), Oct (5), Dec (4)
C1 positive point estimate (any significance)	8	2.2%	Oct (3), Dec (3), May (1), Jul (1)

2.2 Weather on C1 Warming Days

Weather variable	Annual mean	C1 warming days	Difference
Mean air temperature (°C)	24.0	21.2	−2.8
Minimum relative humidity (%)	57.4	72.2	+14.9
Sunshine duration (h)	5.8	1.5	−4.4
Maximum wind speed (m/s)	4.4	5.0	+0.6
Maximum hourly precipitation (mm)	2.3	12.1	+9.8

2.3 Physical Mechanism

C1 apparent warming (8 days, of which 3 significant): All eight days are cold, wet and overcast, with rain or drizzle. Under these conditions transpiration by the vegetation almost ceases, because the stomata close in the absence of sunlight, while the canopy acts as an insulating blanket that suppresses the net longwave loss from the ground to the night sky; vegetated surfaces therefore lose less heat overnight than bare ground. The eight days fall into two groups:

- Winter cold-rain days (3 in December): low temperature, high humidity and near-zero sunshine — typical of the winter cold-front rainfall that affects Shenzhen. The vegetation canopy slows the nighttime cooling of the ground beneath it.

- Typhoon and heavy-rain days (3 in October, 1 in May): after heavy rainfall the soil is saturated, so the dominant cooling pathway shifts from transpiration to direct evaporation, while cloud cover cuts the incoming solar radiation that would otherwise drive a temperature contrast between vegetated and non-vegetated surfaces.

C1 non-significant (23 days): Conditions on these days lie between those of the cooling days and those of the apparently warming days: moderately higher humidity (+6 percentage points), less sunshine (−1.7 h) and stronger wind (+1.2 m/s). Strong winds mix the boundary layer and weaken the temperature contrast between vegetated and non-vegetated surfaces, thereby masking the vegetation signal.

3. C2_pct Anomalous Effects

3.1 Summary Statistics

Category	Days	Share	Dominant Months
C2 significant ($p < 0.05$), all signs	209	57.3%	—
— of which negative (cooling)	**171**	**46.8%**	May (27), Mar (21), Apr (18)
— of which positive (warming)	38	10.4%	Oct (10), Sep (7), Jan (5)
C2 non-significant	156	42.7%	Nov (24), Oct (20), Dec (17)
C2 positive point estimate (any significance)	108	29.6%	Oct (23), Dec (17), Nov (13)

3.2 Weather on C2 Warming Days

Weather variable	Annual mean	C2 warming days	Difference
Mean air temperature (°C)	24.0	22.1	−1.9
Ground surface temperature (°C)	26.6	23.9	−2.7
Atmospheric pressure (hPa)	1005.4	1006.8	+1.4
Sunshine duration (h)	5.8	4.5	−1.3

3.3 Physical Mechanism

C2 apparent warming (108 days, 29.6%; 38 of them significant at $p < 0.05$): These days are strongly concentrated in autumn and early winter, with October–December accounting for 49% of them. Because water has a far larger heat capacity than soil — and, unlike soil, is mixed by convection — it stores large amounts of solar energy over the summer. In autumn the air cools faster than the water, so water bodies release the stored heat to the air above them. Once the water surface is warmer than the land surface, the estimated C2 coefficient reverses sign, changing from negative (summer cooling) to positive (autumn–winter warming).

The associated meteorological signature is: higher atmospheric pressure (+1.4 hPa, reflecting a cold high-pressure system), reduced sunshine (−1.3 h, consistent with the shorter autumn–winter days) and a substantially lower ground surface temperature (−2.7 °C, the land cooling faster than the water).

C2 non-significant (156 days, 42.7%): These days cluster in the transitional seasons, when the water–land thermal contrast is weak and water bodies produce neither clear cooling nor clear warming. Water cover is also very unevenly distributed among the 2500 m buffers: the median C2_pct is only 4.9% and the mean 11.1%, i.e. the distribution is strongly right-skewed, so at many stations little or no water lies within the buffer. This further limits the statistical power available to detect a C2 effect.

4. Correlation: C1/C2 Coefficients vs Weather Variables

Weather variable	r with C1 coefficient	r with C2 coefficient	Physical interpretation
Minimum relative humidity	**+0.50**	+0.03	Higher humidity weakens C1 cooling (transpiration suppressed)
Sunshine duration	**−0.42**	**−0.32**	Stronger sunshine enhances both C1 and C2 cooling
Maximum wind speed	**+0.37**	−0.04	High wind weakens C1 cooling (boundary-layer mixing)
Maximum air temperature	+0.10	**−0.30**	Higher temperature strengthens C2 cooling (water evaporation)
Ground surface temperature	+0.22	**−0.32**	Higher ground surface temperature strengthens C2 cooling
Atmospheric pressure	**−0.32**	+0.16	High pressure (cold air mass) weakens C1 cooling

5. Conclusions

1. The C1 vegetation cooling signal is robust: the coefficient is statistically significant on 93.7% of days and negative on 92.9% (339 of 365). Only 8 days (2.2%) yield a positive point estimate, and just 3 of those are statistically significant; all 8 have a clear physical explanation — the insulating effect of the canopy — so they do not undermine the overall conclusion that vegetation cools the air.
2. The C2 water effect is weaker and strongly seasonal: the coefficient is significant on 57.3% of days, but negative on only 171 days (46.8%) — mainly in spring and summer — and positive on 38 days (10.4%), mainly in autumn and winter. The seasonal cycle of heat storage and release reverses the sign of the coefficient, so C2 cannot be relied upon as a year-round cooling indicator.
3. Key meteorological drivers: sunshine duration, humidity and wind speed are the variables most strongly associated with the strength of the C1 cooling effect ($|r| > 0.35$). Hot, dry and calm days give the strongest vegetation cooling; wet, cold, windy and overcast days give the weakest, occasionally reversing to apparent warming.
4. Applicability of the model: on 97.8% of days (357 of 365, i.e. all but the 8 days with a positive point estimate, of which only 3 are statistically significant) the daily C1 signal points in the expected direction — cooling, or at least no warming — and it breaks down only under extreme weather. At the annual scale, aggregating over all 365 days removes these single-day anomalies, and the mean coefficient of −0.0128 °C per percentage point of vegetation cover is robust.

S3: Built-up Area Temperature vs Total Electricity Consumption Regression Analysis Results

2021 Shenzhen | 365 daily observations | Quadratic model + day-type dummies

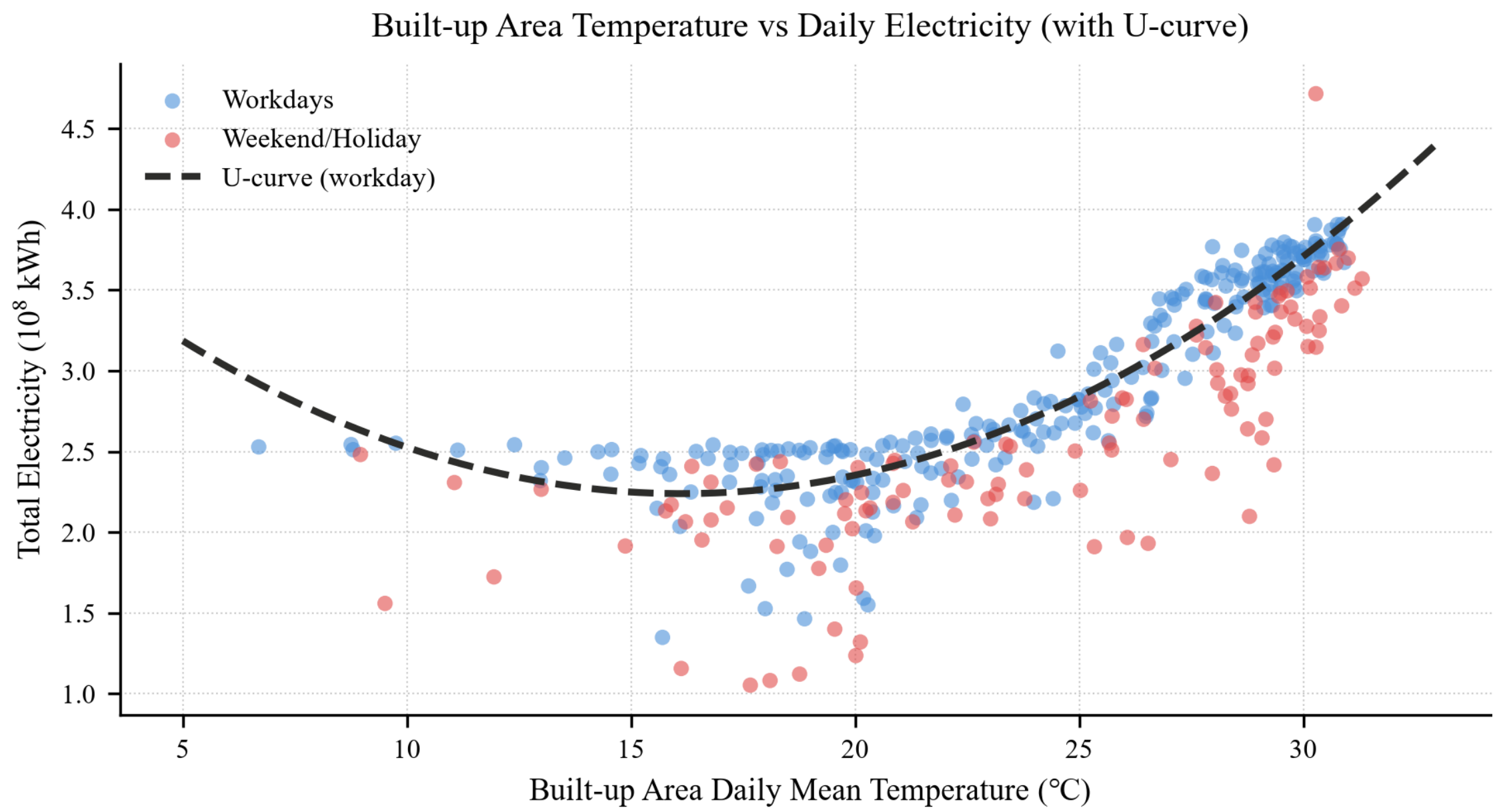

1. Model Specification

$$Elec = \beta_0 + \beta_1 \cdot T + \beta_2 \cdot T^2 + \beta_3 \cdot SpringFest + \beta_4 \cdot OtherHol + \beta_5 \cdot Makeup + \beta_6 \cdot Weekend + \varepsilon$$

2. Regression Results

Variable	Coef	SE	t	p	
Intercept	**+4.2271**	0.1852	+22.8	**8.61e-72**	***
T (built-up temp)	**-0.2465**	0.0170	-14.5	**1.05e-37**	***
T^2	**+0.0076**	0.0004	+20.2	**3.87e-61**	***
Spring Festival	**-1.1046**	0.0891	-12.4	**1.33e-29**	***
Other Holidays	**-0.6565**	0.0494	-13.3	**4.80e-33**	***
Makeup Workdays	-0.2248	0.0882	-2.5	1.12e-2	*
Weekends	**-0.2236**	0.0291	-7.7	**1.45e-13**	***

Statistic	Value
R^2 / Adj R^2	0.88 / 0.878
F-test	F=436, p=2.91e-161
Observations	365 days
RMSE	0.228 × 10^8 kWh (8.1% of daily mean)
MAE	0.168 × 10^8 kWh (6.0% of daily mean)

> All variables significant at $p<0.05$. F=436 ($p \approx 0$). Adj R^2=0.878 indicates the model explains ~88% of daily electricity variation.

3. Fit

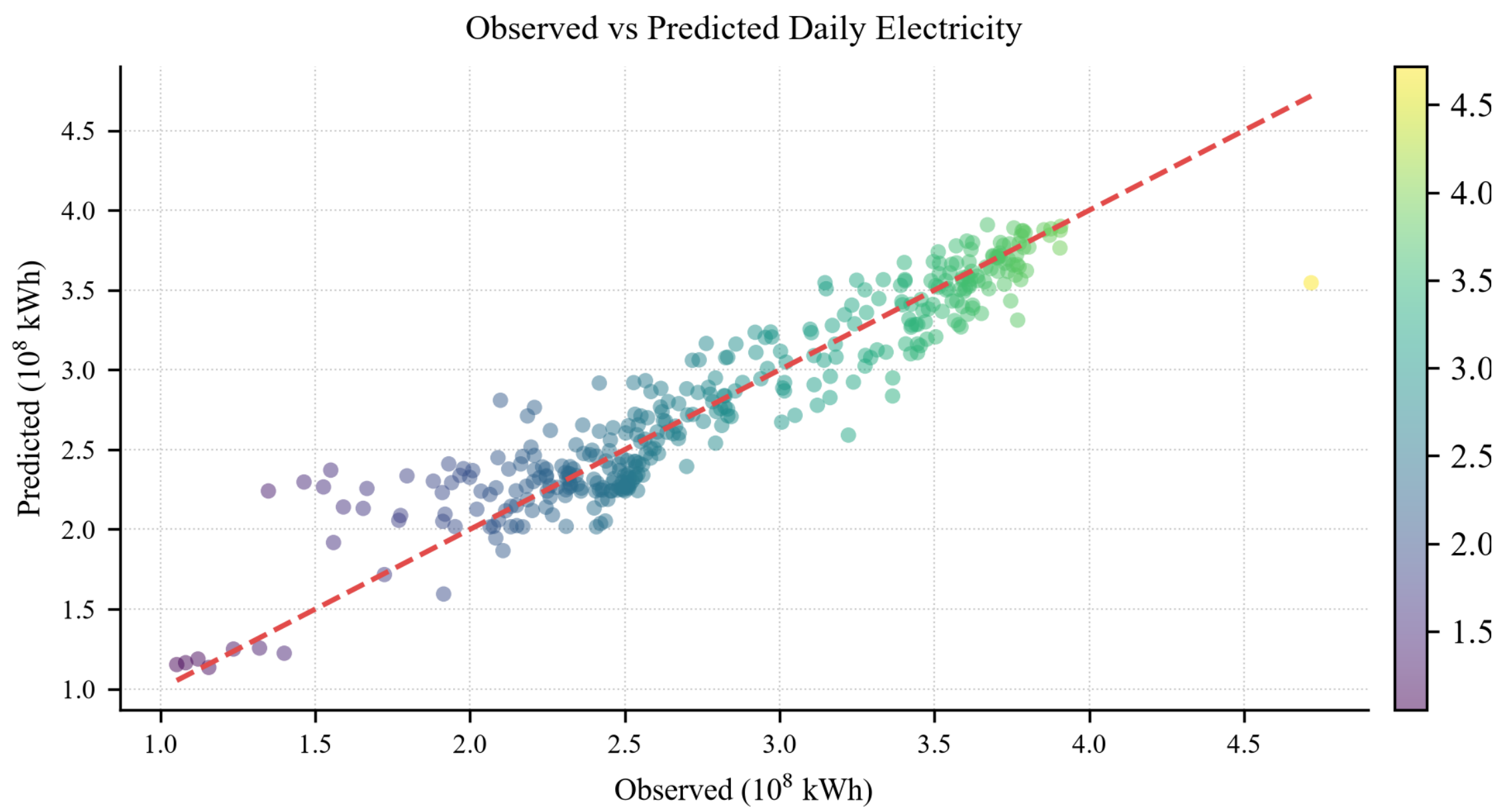

4. Physical Interpretation of Temperature-Electricity Function

4.1 U-shaped Curve and Comfort Temperature

The quadratic function opens upward, indicating a minimum electricity consumption point (comfort temperature):

$$\mathbf{d(Elec)/dT = -0.2465 + 2\times0.0076\times T = 0 \rightarrow T_{opt} = 16.1°C}$$

At **16.1°C**, Shenzhen's built-up area requires neither cooling nor heating, with electricity at its annual minimum (~2.24 × 10^8 kWh on workdays).

4.2 Marginal Effect by Temperature

Temperature	Marginal Increase	Interpretation
10°C	**-937 × 10^4 kWh/°C**	Low-temperature zone, theoretical extrapolation (rare in Shenzhen)
15°C	**-173 × 10^4 kWh/°C**	Near comfort zone, weak negative effect
20°C	**+591 × 10^4 kWh/°C**	AC begins operation, positive sensitivity
25°C	**+1355 × 10^4 kWh/°C**	Cooling load rises significantly
30°C	**+2119 × 10^4 kWh/°C**	Extreme heat: each 0.1°C cooling saves ~211×10^4 kWh citywide

5. Day-Type Dummy Variable Effects

Day Type	Days	Effect (10^8 kWh)	Interpretation
Regular Workday (baseline)	243	—	Reference group
Spring Festival	7	**-1.10**	Near-empty city, electricity halved

Day Type	Days	Effect (10^8 kWh)	Interpretation
Other Statutory Holidays	24	**-0.66**	Offices closed, manufacturing reduced
Makeup Workdays	7	-0.22	Partial resumption, still below normal
Regular Weekends	84	**-0.22**	Offices fully closed, factories partially running

6. Model Diagnostics

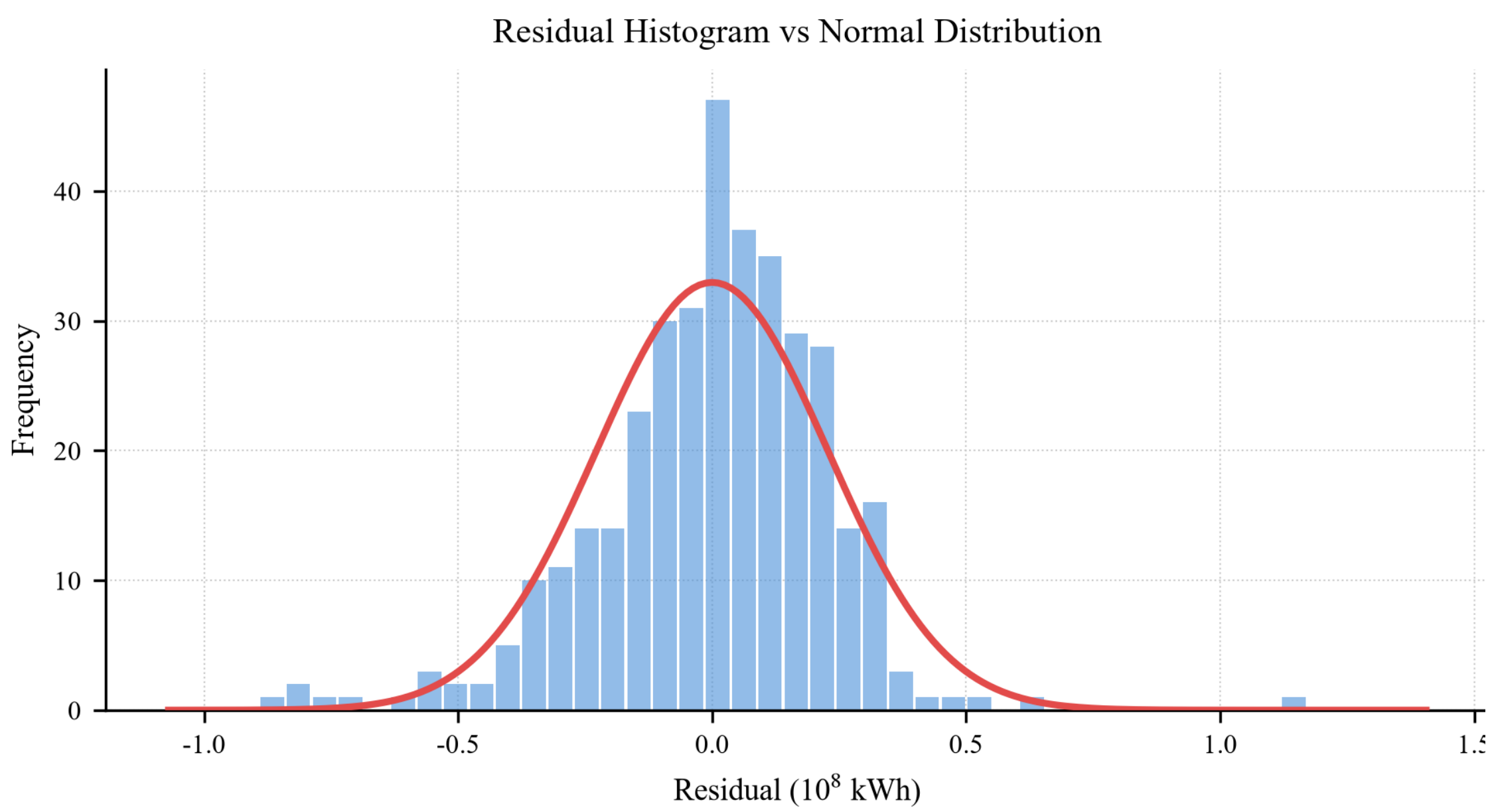

6.1 Residual Normality

Jarque-Bera = 141, p=2.69e-31—**normality formally rejected**. Skewness = -0.39 (slight left skew), kurtosis = +2.94 (fat tails). With N=365, the Central Limit Theorem ensures valid coefficient inference. Fat tails mainly driven by extreme days such as Spring Festival.

6.2 Autocorrelation

Durbin-Watson = **1.039**. Close to 2; residuals are approximately white noise—the quadratic function adequately captures daily temperature-electricity dynamics.

6.3 Heteroskedasticity and Multicollinearity

Breusch-Pagan LM=14, p=0.025. Temperature and its quadratic term exhibit structural collinearity (VIF≈41, unavoidable in quadratic regression). Day-type dummies show negligible collinearity (VIF<1.3).

6.4 Prediction Accuracy

Metric	Value
RMSE	0.228×10^8 kWh (8.1% of daily mean)
MAE	0.168×10^8 kWh (6.0% of daily mean)

Metric	Value
90% Residual Interval	[-0.369, +0.305] × 10^8 kWh

7. 10-Fold Cross-Validation

To evaluate generalization, 10-fold CV was performed (shuffle, seed=42). Each fold trains on ~328 days and tests on ~37 days.

Fold	Train	Test	R^2	RMSE	MAE
1	328	37	0.885	0.237	0.173
2	328	37	0.903	0.207	0.166
3	328	37	0.905	0.17	0.135
4	328	37	0.914	0.175	0.136
5	328	37	0.834	0.236	0.156
6	329	36	0.869	0.228	0.182
7	329	36	0.91	0.204	0.163
8	329	36	0.798	0.296	0.226
9	329	36	0.816	0.32	0.213
10	329	36	0.881	0.228	0.182

Metric	Full Sample	10-Fold CV	Δ
R^2	0.88	**0.873**	+0.007
RMSE (10^8 kWh)	0.228	**0.234**	+2.8%
MAE (10^8 kWh)	0.168	**0.173**	+2.8%

> CV R^2=0.873, only 0.007 below full-sample. RMSE on unseen data is 8.3% of daily mean, nearly identical to training (8.1%). No overfitting. Folds 8–9 have lower R^2 (~0.81) due to Spring Festival/holiday days concentrated in the test set.

7.1 Daily Prediction Accuracy vs Annual Cooling Savings

The daily prediction RMSE of ~8% means a 1% cooling saving (~0.028 × 10^8 kWh) is well below the noise floor—**precise single-day savings prediction is infeasible**. However, this does not preclude annual scenario estimation:

The quadratic coefficient (β_2=+0.0076, SE=0.0004, t=20.2) is estimated from all 365 days jointly. Its confidence interval is far narrower than the single-day prediction residual. At 30°C, the marginal effect is ~0.21 × 10^8 kWh/°C with 95% CI ≈ ±0.03—the effect is over 7 times its standard error, **clearly distinguishable**.

Daily prediction accuracy of 8% does not equal 8% accuracy for the marginal effect. The model estimates a U-shaped curve's position and curvature from 365 observations; the curve parameters are far more precise than single-point predictions. Annual total: assuming 200 cooling days and 0.1°C cooling, annual savings ≈ 4.2 × 10^8 kWh, with cumulative error well below 1%. **Annual cooling savings estimation is fully feasible.**

8. Conclusions

1. Built-up area temperature and electricity consumption exhibit a **clear U-shaped quadratic relationship**, R^2=0.88, all variables $p<0.05$.
2. Shenzhen's electricity comfort temperature is approximately **16.1°C**; above this, cooling load accelerates nonlinearly.
3. At 30°C, each 0.1°C increase costs ~**211×10^4 kWh**; cooling by 0.1°C citywide could save hundreds of millions of kWh annually.

4. 10-fold CV R^2=0.873, good generalization. Daily RMSE ~8% does not compromise annual savings estimation—coefficient precision is determined by all 365 observations jointly.

5. Spring Festival effect (-1.10) is approximately **5 times** the weekend effect (-0.22).

6. DW=1.039, residuals approximately white noise—coefficient inference is reliable and suitable for annual savings estimation.

Figures

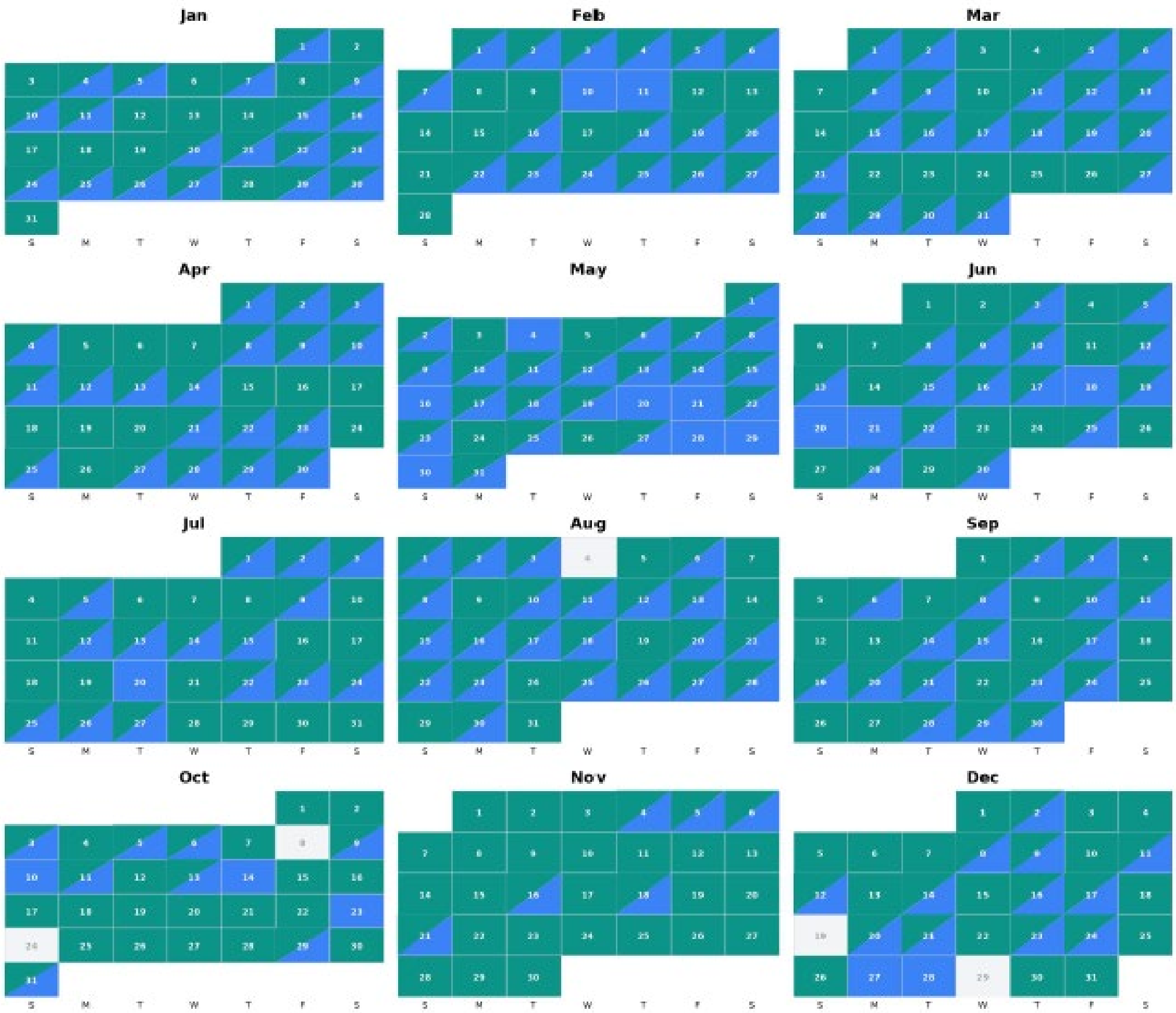

Figure S1. Daily significance calendar of vegetation and water cooling effects in 2021. Of 365 days, at least one variable was significant ($p < 0.05$) on 360 days (98.6%): vegetation only (green) was significant on 151 days, water only (blue) on 18 days, both were significant (diagonal green/blue split) on 191 days, and neither was significant (gray) on only 5 days.

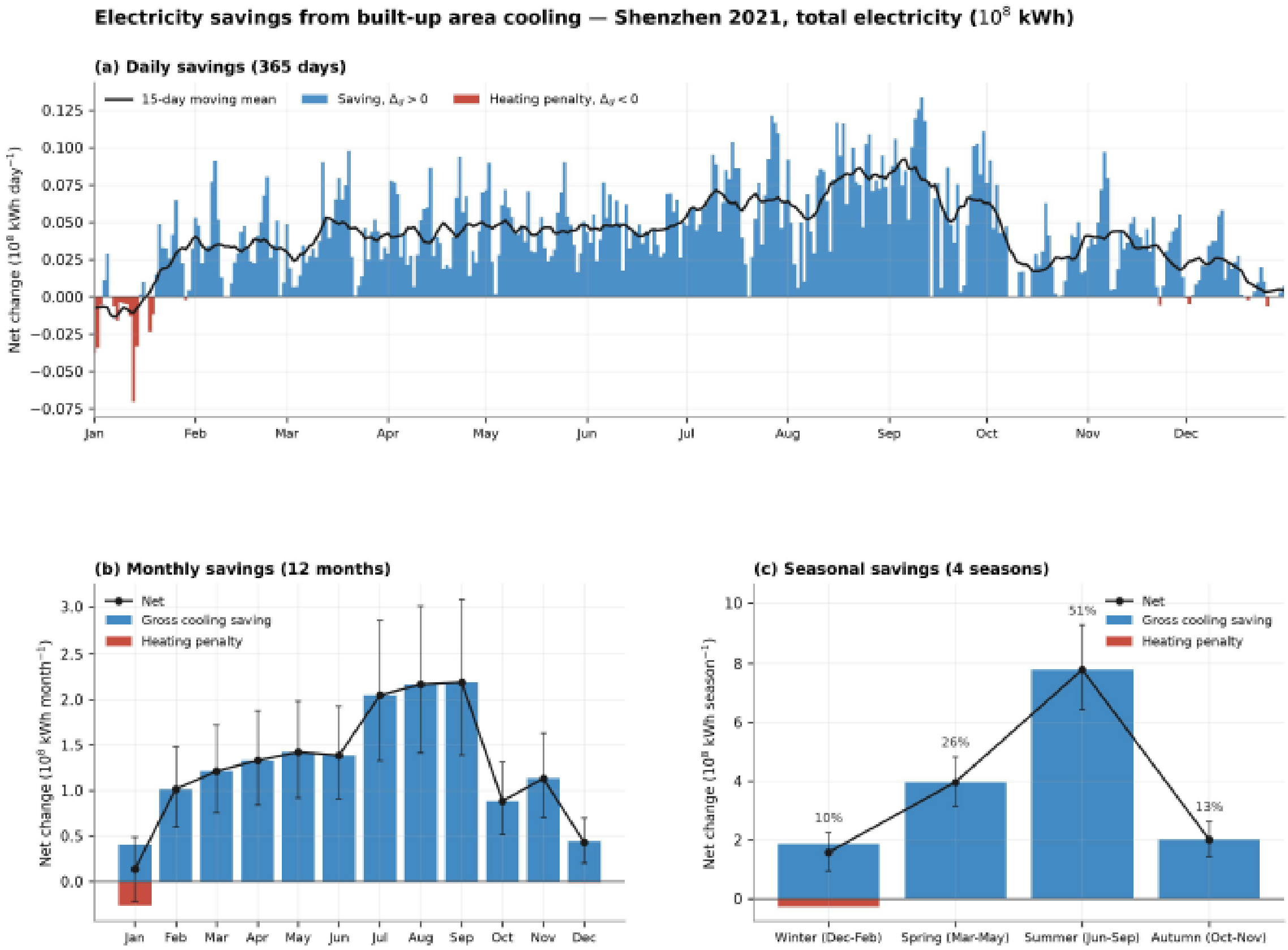

Figure S2. Temporal distribution of daily total electricity savings from built-up area cooling in Shenzhen, 2021. Savings are estimated day by day as the difference in predicted electricity use between the observed temperature and the simulated cooled temperature, using the fitted temperature–electricity response; day-type effects cancel in this difference and therefore do not affect it. In all panels, blue bars are days on which cooling reduces electricity use and red bars are days on which cooling slightly increases it because it adds to heating demand. (a) Daily savings; the black line is a 15-day moving mean. (b) Monthly and (c) seasonal savings; black markers show the net saving for each month or season with its 95% confidence interval. All four seasons show a net saving, with summer contributing the largest share and winter the smallest; the winter heating penalty is small and offsets only a negligible part of the annual gain. Savings are thus spread across the whole year rather than concentrated in a few hot days.
